# Hospital Notes

**Published:** 1894-10

**Authors:** 


### Hospital Notes.

THE operating amphitheater of the hospital of the Medical College of Virginia, at Richmond, has been remodeled and supplied with all the latest aseptic operating furniture and apparatus. This department is presided over by a trained nurse, whose sole duty is to care for the operating-room, prepare patients for operation and assist at operations.

Two vacancies will occur on the house-staff of the Buffalo General Hospital, November 1, 1894. Term of service, one year; six months on medical and surgical divisions respectively. Candidates must be graduates in medicine. For full particulars address Dr. C. Diehl, secretary of staff, 32 West Genesee street, Buffalo, N. Y.

